# Bare Earth’s Surface Spectra as a Proxy for Soil Resource Monitoring

**DOI:** 10.1038/s41598-020-61408-1

**Published:** 2020-03-10

**Authors:** José A. M. Demattê, José Lucas Safanelli, Raul Roberto Poppiel, Rodnei Rizzo, Nélida Elizabet Quiñonez Silvero, Wanderson de Sousa Mendes, Benito Roberto Bonfatti, André Carnieletto Dotto, Diego Fernando Urbina Salazar, Fellipe Alcântara de Oliveira Mello, Ariane Francine da Silveira Paiva, Arnaldo Barros Souza, Natasha Valadares dos Santos, Cláudia Maria Nascimento, Danilo Cesar de Mello, Henrique Bellinaso, Luiz Gonzaga Neto, Merilyn Taynara Accorsi Amorim, Maria Eduarda Bispo de Resende, Julia da Souza Vieira, Louise Gunter de Queiroz, Bruna Cristina Gallo, Veridiana Maria Sayão, Caroline Jardim da Silva Lisboa

**Affiliations:** Department of Soil Science, Luiz de Queiroz College of Agriculture, University of São Paulo, GeoCis (Geotechnologies in Soil Science Group (https://esalqgeocis.wixsite.com/english), Ave Padua Dias 11, Piracicaba, Sao Paulo 13418-900 Brazil

**Keywords:** Environmental sciences, Environmental impact

## Abstract

The Earth’s surface dynamics provide essential information for guiding environmental and agricultural policies. Uncovered and unprotected surfaces experience several undesirable effects, which can affect soil ecosystem functions. We developed a technique to identify global bare surface areas and their dynamics based on multitemporal remote sensing images to aid the spatiotemporal evaluation of anthropic and natural phenomena. The bare Earth’s surface and its changes were recognized by Landsat image processing over a time range of 30 years using the Google Earth Engine platform. Two additional products were obtained with a similar technique: a) Earth’s bare surface frequency, which represents where and how many times a single pixel was detected as bare surface, based on Landsat series, and b) Earth’s bare soil tendency, which represents the tendency of bare surface to increase or decrease. This technique enabled the retrieval of bare surfaces on 32% of Earth’s total land area and on 95% of land when considering only agricultural areas. From a multitemporal perspective, the technique found a 2.8% increase in bare surfaces during the period on a global scale. However, the rate of soil exposure decreased by ~4.8% in the same period. The increase in bare surfaces shows that agricultural areas are increasing worldwide. The decreasing rate of soil exposure indicates that, unlike popular opinion, more soils have been covered due to the adoption of conservation agriculture practices, which may reduce soil degradation.

## Introduction

Soils are directly related to global issues, such as food supply, water security and climate regulation^1–4^. Considering that soil health is easily disturbed by modifications of physical, chemical and biological conditions, soil management must be carefully delineated and constantly monitored^5^. The high occurrence of bare surfaces in agricultural areas may increase soil degradation, influencing soil health and affecting important ecosystem functions^6^. Surface exposure triggers a sequence of events, which may result in soil erosion^7^, contamination^8^, desertification^9^, salinization^10^, acidification^11^, compaction^12^, biodiversity loss^13^, nutrient depletion^14^, and loss of soil organic carbon (SOC)^15^. Despite the need to preserve soil ecosystems for future generations, we will still need to feed 9.7 billion people by the middle of this century^16^. To date, the total area of worldwide crop and pasture lands is estimated at 49 million km^2^, which represents approximately 30% of the total global land area^17^. The world’s future demand for water, energy and food is closely related to the intensification of agriculture and the preservation of ecosystems.

Sustainable land use requires data-driven policymaking and management strategies, which are currently hampered by the high cost and time-consuming characteristics of soil data acquisition^2^. The remote sensing (RS) technique is an indispensable tool for multitemporal and spatial data acquisition, as it enables the collection of soil information at global scales^18^. Although RS is an important source of information, its use is more frequent in the assessment of landscape vegetation cover. Few studies address uncovered surfaces, and mostly are conducted at national scales^19–21^. Therefore, we developed a big-data-mining method to perform a planetary-scale geospatial analysis of surfaces. The method can identify the global soil spatial variability and describe the frequency of bare surfaces over time. Temporal changes in bare surfaces have been applied to identify shifts in soil management strategies (e.g., till to no-till systems) over the last three decades. Such knowledge and understanding enable users to identify highly threatened areas around the globe, and this method can be used to reveal locations where soil management conditions have been improved or not.

## Results and Disscussion

### Earth’s bare surface detected from space

**Figure 1:**
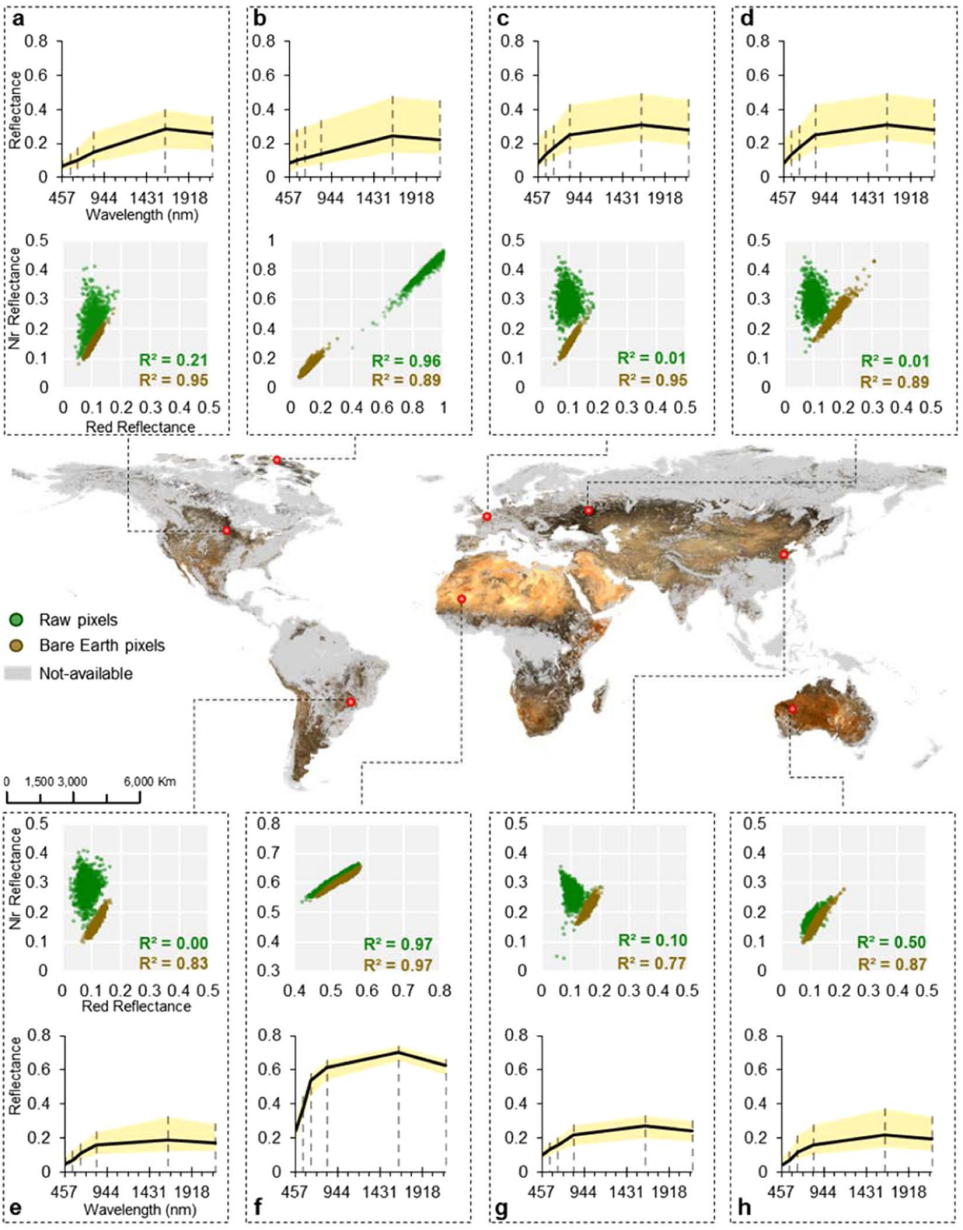
Earth’s bare surface reflectance. True colour composition (red: 630–690 nm, green: 520–600 nm, blue: 450–520 nm) at 250 m resolution retrieved through Landsat series observations from 1985 to 2015 (Landsat 4, 5, 7 and 8 images courtesy of the U.S. Geological Survey). Soil line (red and NIR reflectance) and spectral signatures (blue to SWIR_2_ ranges) constructed from sampling bare (processed) and raw (unprocessed) pixels in 50 km^2^ areas at different locations of the world. Raw data were acquired by using Landsat 8 surface reflectance median composites from 2017 to 2019: (**a**) North America; (**b**) Arctic Desert; (**c**) Europe; (**d**) Central Asia; (**e**) South America; (**f**) Sahara Desert; (**g**) East Asia; and (**h**) Australian desert.

The monitoring of Earth’s bare surfaces is limited by many factors, including highly vegetated landscapes, short exposure time gaps in crop fields and spectral degradation^22^. A single satellite image detects only fragments of bare surfaces, and their measurements are highly influenced by external factors, such as surface roughness, moisture levels, and undefined material deposition^23,24^. Therefore, an adequate synoptic view of the surface can be achieved only using multitemporal composite images^23^. Our data mining method seeks to minimize these impacts and provide a reasonable representation of Earth’s bare surfaces using efficient soil classification rules and a robust aggregation approach. We generated a product called the Synthetic Soil Image (SYSI) using the Geospatial Soil Sensing System (GEOS3)^23^. The SYSI is the result of the aggregation of multiple bare surface (BS) fragments mapped from 1985 to 2015 by Landsat satellites (Fig. 1). The SYSI’s capacity to retrieve Earth’s BS is almost twofold when compared to global land cover mappings, such as the annual land cover mapping of Moderate Resolution Imaging Spectroradiometer^25^ (MODIS) and finer resolution maps generated using Landsat^26^. Our product covered 32% of the Earth’s surface (Fig. 1), comprising mostly bare lands (approximately 15% when Antarctica and Greenland are included), which is in agreement with the literature^25,26^. The remaining 17% is composed of agricultural areas (the value was close to 12.6% in 2017^27^), abandoned fields or naturally bare surfaces outside arid regions.

The spectral pattern retrieved from agricultural BS is related, in many cases, to soil surfaces exposed during land conversion and soil tillage practices. The spatial-spectral patterns, therefore, may be directly related to soil components such as mineralogy, granulometry and carbon^28^. The analysis of the SYSI true colour composition (red: 630–690 nm; green: 520–600 nm; blue: 450–520 nm) reveals many properties based on the colour differences (Fig. 1). In terms of soils, brighter locations in the true colour composition are most likely related to higher quartz proportions^29^. When quartz and SOC contents are low and/or moist and iron forms (which absorb energy) are proportionally higher, the colour becomes darker^29^. The extensive detection of bare arid lands, e.g., deserts in the north-western United States, South America (Atacama), southern Africa (Kalahari) and northern Africa (Sahara), Asia (Gobi) and Australia (West Desert), provides great evidence of SYSI’s potential to retrieve bare surfaces (Fig. 1).

The reliability and quality of the image were evaluated by site-specific observations of surface reflectance (Fig. 1a–h), which presented topsoil data that contrasted to the common landscape patterns (such as vegetation, dry vegetation, water, and snow). The evaluation was performed using a scatter plot of spectral bands, commonly termed soil line analysis^20^. Common landscape patterns have a distinct scattered distribution between the red and infrared bands. This difference is associated with the absorption in the visible ranges by pigments and scattering/reflectance effects of vegetation structures in the infrared bands^22^, which implies a spread distribution between these two bands. Soils, however, have a linear relationship due to the increase in reflectance from visible to infrared bands, which is related to the scattering effects of particles and minerals from the surface matrix^24^. All evaluated scatter plots (Fig. 1a–h) presented differences between typical patterns (raw pixels) and soils (bare surface pixels), supporting the premise that our data mining system identifies soils in satellite images.

Additionally, the evaluation of spectral signatures can assist in obtaining information about object properties. There is a strong relationship between soils and radiant energy, which has been systematically proven at regional (United States)^30^ and global scales (World’s Spectral Library)^28^ using laboratory spectral datasets. Although absorption features of high-resolution spectra are more adequate for identifying soil constituents^29^, multispectral reflectance can still be used to identify main Earth characteristics, such as describing shape and intensity patterns of spectral curves (line chart in Fig. 1a–h). The spectral increase from blue to near-infrared can reveal the effects of SOC, iron oxides and other opaque minerals^22^. Spectral curves of temperate zones such as North America (Fig. 1a) and the Arctic Desert (Fig. 1b) have a concave shape affected by high amounts of SOC. Conversely, in the Sahara Desert (Fig. 1f), the presence of reflective minerals (e.g., quartz) results in a convex spectral shape. Other regions have a more linear shape due to mixed effects of soil properties, e.g., in China (Fig. 1g). Iron-rich soils from tropical regions, such as those from South America and Australia (Fig. 1e,h), have a higher reflectance in the red band.

The soil reflectance could also provide particle size information due to scattering and reflection effects^22^. Soils with low reflectance contain high amounts of opaque minerals or are predominantly formed by finer particles (clay: <2 μm and silt: between 2 μm and 50 μm). This effect was observed in North America and the Arctic Desert (Fig. 1a,b) and in clayey soils from South America (Fig. 1e), China (Fig. 1g) and Australia (Fig. 1h). The medium-textured soils of Europe and Central Asia (Fig. 1c,d) had intermediate reflectance intensity, while the quartz-rich surface of the Sahara Desert (Fig. 1f) had the highest intensity. Thus, remotely sensed spectra have an intimate relationship with soil attributes and are useful for mapping carbon^31^, clay^32^, and cation exchange capacity^33^.

### Enhancing global soil resource monitoring by bare surface imaging

The SYSI dataset not only provides a representation of BS but can also be related to lithological and pedological variability, carbon-rich pools, and biome changes. Lithologic classes (Extended Data Fig. 1a) from the African continent showed a transition from sedimentary to metamorphic rocks from north to south^34^, which were identified using a false colour composition (red: 1550–1750 nm, green: 760–900 nm, blue: 630–690 nm), changing from bright magenta to purple shades (Extended Data Fig. 1b). Volcanic and unconsolidated materials in South America, West Africa, India, and Central Asia had a strong purple shade (Site 1, Extended Data Fig. 1b). Conversely, sedimentary materials (Site 2; Extended Data Fig. 1b) presented a magenta shade, reaching a bright hue over the main deserts.

The Lithological Global Map and the SYSI had a correlation of 0.22, which represents a meaningful value when considering the global scale. The spatial resolution of the SYSI (250 m) and the capacity to retrieve spectral features from Earth’s surface could be useful for improving lithological maps around the world. An example of this potential is shown in Australia (Site 3, Extended Data Fig. 1b), where volcanic rocks appear to be more visible than the unconsolidated sediments indicated by the lithological map.

The contrast among lighter and darker surfaces may be related to SOC content^35^ and, consequently, to some of the greatest SOC pools on Earth (Extended Data Fig. 2a,b). The SYSI indicated that dark bare surfaces, such as those in southern Russia (Extended Data Fig. 2a), are often related to a higher SOC content (Extended Data Fig. 2a), which was confirmed by the negative correlation of −0.35. The same pattern was observed in Asia (site 1), Central Africa (site 2), North Africa and the Central East (site 3) (Extended Data Fig. 2a).

The SYSI also has great potential for enhancing pedological maps due to the strong connections between reflectance from visible to near-infrared and different soil classes^36^ (Extended Data Fig. 3a,b), which reached a correlation of 0.61. Site 1 indicates areas with Regosols, where quartz-rich areas from the Sahara Desert are detected in the SYSI. Site 2 presents a transition from Kastaznozems to Chernozems to Solonchaks due to an increase in SOC content, which was also observed by a change in shades from bright to dark (Extended Data Fig. 3a). A very similar delineation between Gleysols to Kastaznozems and Yemasols was observed at site 3, since this region is an area of wetland agriculture (mainly rice). In South America (Extended Data Fig. 4a), areas with volcanic rocks are closely related to Ferralsols, displaying a darker red coloration in the true colour composition and holding a high amount of SOC content. Regosols and Yermosols in arid regions (rich in quartz) present a higher albedo and brighter features (Extended Data Fig. 4a). In southern Brazil, high basaltic extrusions resulted in Ferralsols (clayey and iron rich soils), which have a typical dark purple pattern in the false colour composition of the SYSI (Extended Data Fig. 4b). Wetland soils (e.g., Gleysols) can also be discriminated in the image, such as in Southeast Asia (Extended Data Fig. 4c), where the reduced iron forms combined with the higher moisture resulted in a distinct spectral pattern (Extended Data Fig. 4d). A sudden change in tree canopy cover, organic carbon content^37,38^, and pedological classes can be detected in the transition between the Sahara Desert and the Sub-Saharan region, where more vegetated areas appear (Extended Data Fig. 4d).

### Human-induced changes in land use based on bare surface monitoring: impacts on Earth’s soil resources

**Figure 2:**
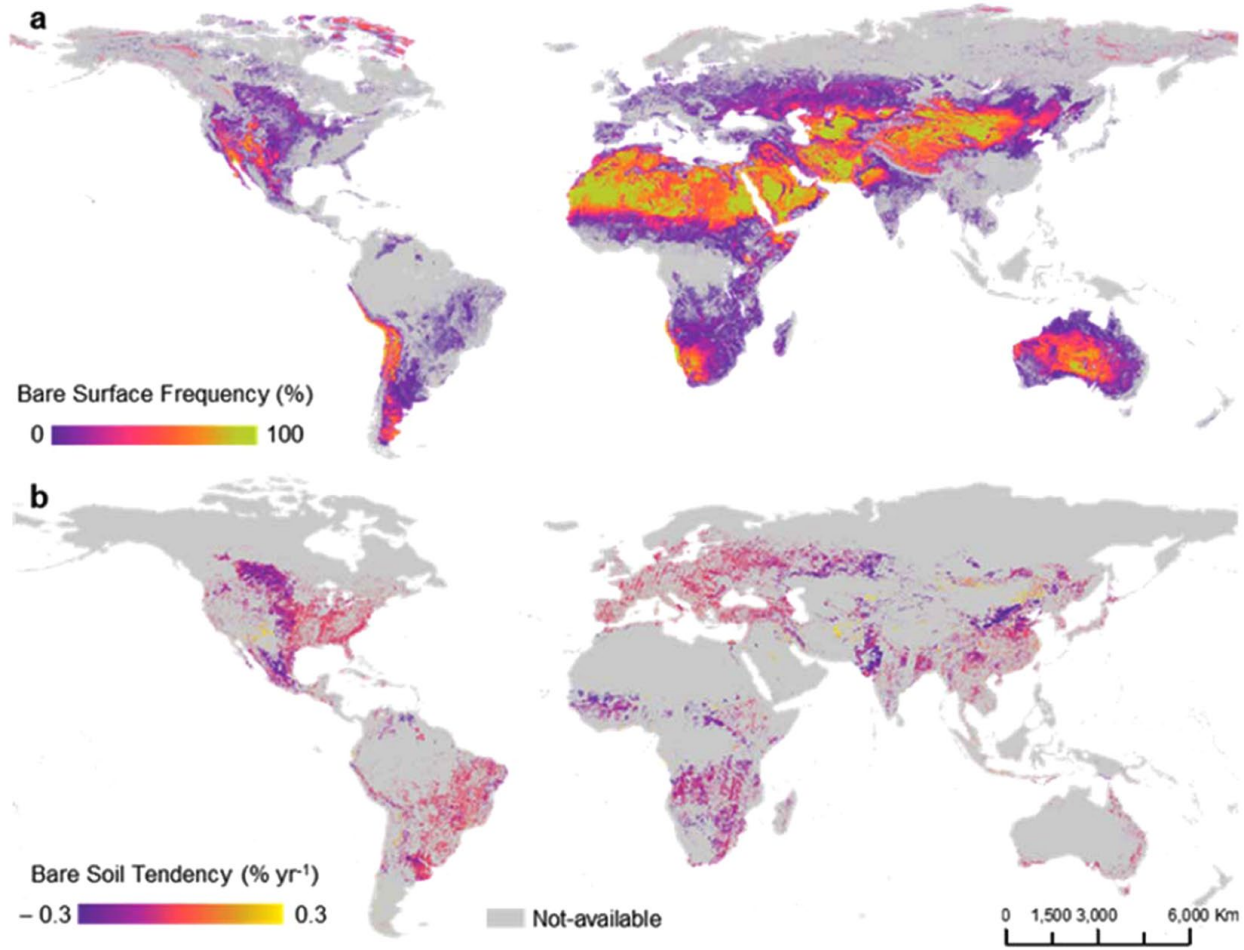
Earth’s bare surface analysis from 1985 to 2015. (**a**) Bare surface frequency (number of times the pixel was bare during the period); (**b**) bare soil tendency (tendency to be bare during the period), which were constrained by the MODIS Land Cover Yearly Mapping reference map generated in 2017^69^. (Landsat 4, 5, 7 and 8 images courtesy of the U.S. Geological Survey).

The SYSI not only provided crucial data about soils worldwide but also provided temporal evidence of human-induced changes in land use/management (Fig. 2a,b). Most importantly, such data provided a complementary perspective in analysing land-use changes. Instead of focusing on average annual spectral patterns, it was possible to emphasize the spatial extent and frequency of Earth’s bare surfaces. Thus, we proposed a by-product of the BS image, called hereafter Earth’s bare surface frequency (BSF) (Fig. 2a). This image represents where and how many times a single pixel was detected as bare using the Landsat series. The frequency of bare pixels is higher in deserts, which was already expected due to the edaphoclimatic conditions of these areas (Fig. 2a). In general, the BSF decreased over agricultural areas in South America, Africa, the Australian coast and North America. The BS on the global scale (Fig. 2a,b) provides strong evidence of land disturbance, contributing to the discussion of its consequences, such as erosion^39^ and changes in energy balance/climate/emissivity^40,41^, temperature^42^, moisture^43^, SOC stocks^1^ and microorganism populations^44^. In addition, an increase in BSF may promote greater weed growth^45^, which may intensify pesticide use^46^ and, consequently, groundwater contamination^47^.

**Figure 3:**
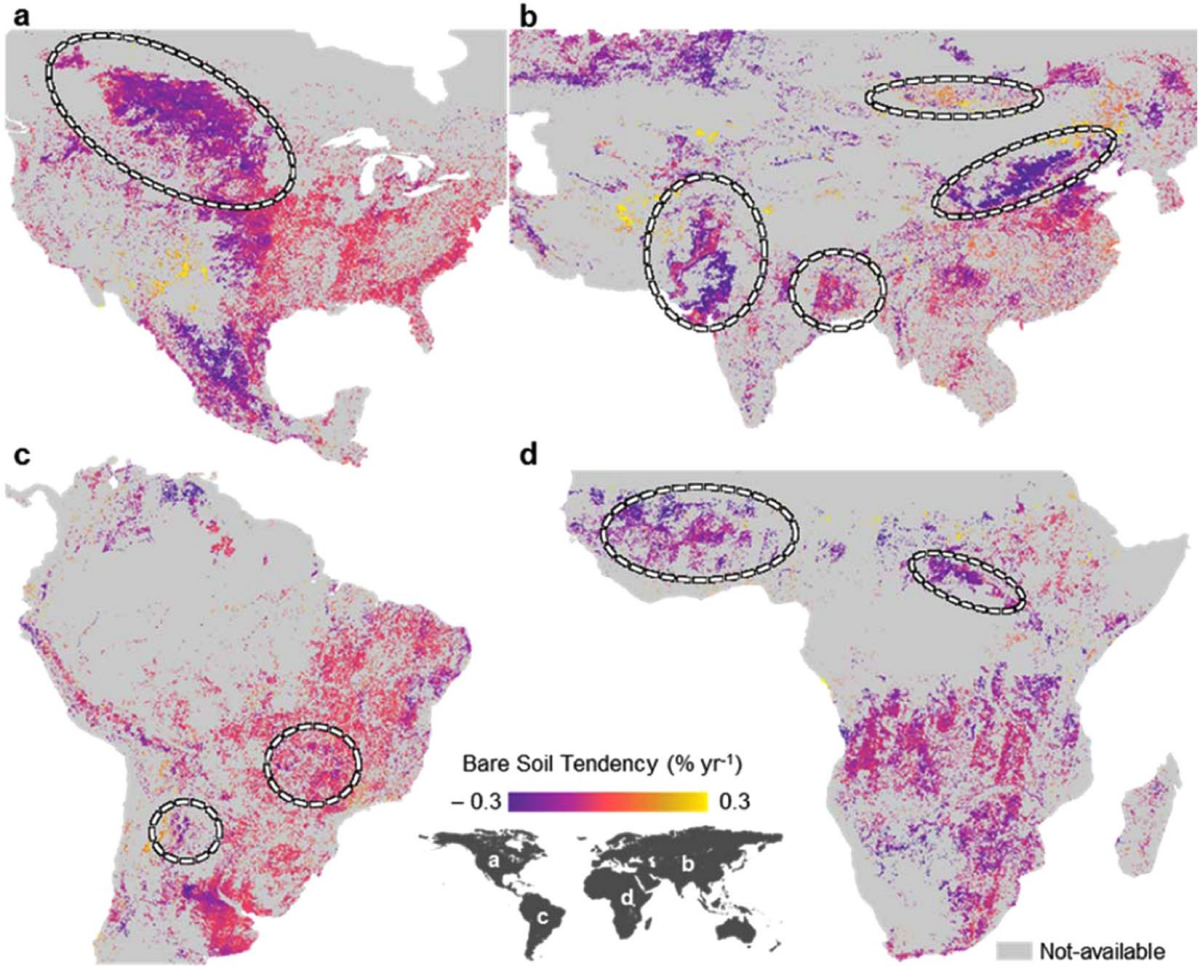
Bare soil tendency. Areas with statistical significance in terms of being bare over 35 years were evaluated in different regions of the globe. Specific locations in the globe and its relationship with the bare soil tendency (BST); (**a**) United States; (**b**) Asia; (**c**) South America; and (**d**) Africa. (Landsat 4, 5, 7 and 8 images courtesy of the U.S. Geological Survey).

Earth’s bare soil tendency (BST) was calculated between 1985 and 2015 to focus on soil resource use from agriculture (Fig. 2b). The SYSI was able to identify 95% of bare soils within agricultural areas around the globe. Some studies have reported similar coverage of bare areas on agricultural lands^20^. The BST could be used to enhance our understanding of what has happened to the soils in the last 30 years. In general, the tendency has shown a decrease in soil exposure (negative values, Fig. 2b) in most countries. The global trend of exposed surface decreased by approximately 4.8%. In the Central eastern United States and southern Canada and some spots in Asia (Fig. 3a,b), there was a smooth downward trend in the BST (purple colour). Some spots indicated an upward trend in the BST, such as in Central Brazil and Central Africa (Fig. 3c,d). In the Central eastern United States and some spots in Europe (Fig. 3a,b), there was a smooth trend in the BST (near zero, Fig. 2b).

An expressive reduction in the BST was observed in the Great Plains in North America (Fig. 3a), which was most likely related to the conversion of agricultural areas to pasture (10–26% decline in agricultural lands). On the Asian continent, there were many areas with significant changes, such as in northern India and eastern China (Fig. 3b). In these locations, the BSF decreased in recent decades. These changes were related to an increase in irrigation systems^48^, which allowed farmers to cultivate throughout the year. In north-eastern China and Central Mongolia, there was a higher increase in the BST (0.3% yr^−1^), which was similar to that observed by Song *et al*.^48^. According to these authors, such areas exhibited a large decrease in short vegetation and an increase in BS related to land degradation. In South America, many areas have undergone changes in recent decades, but one of the most significant is the Brazilian agriculture frontier, located near the Amazon biome. This area of southern Amazon forest at the fringe of the Amazon biome experienced deforestation and land-use intensification^49^ (Fig. 3c), which caused considerable land degradation. This process was detected by an extremely high increase in the BST (0.3% yr^−1^). On the African continent, most of the areas presented a negative tendency (Fig. 3d).

**Figure 4:**
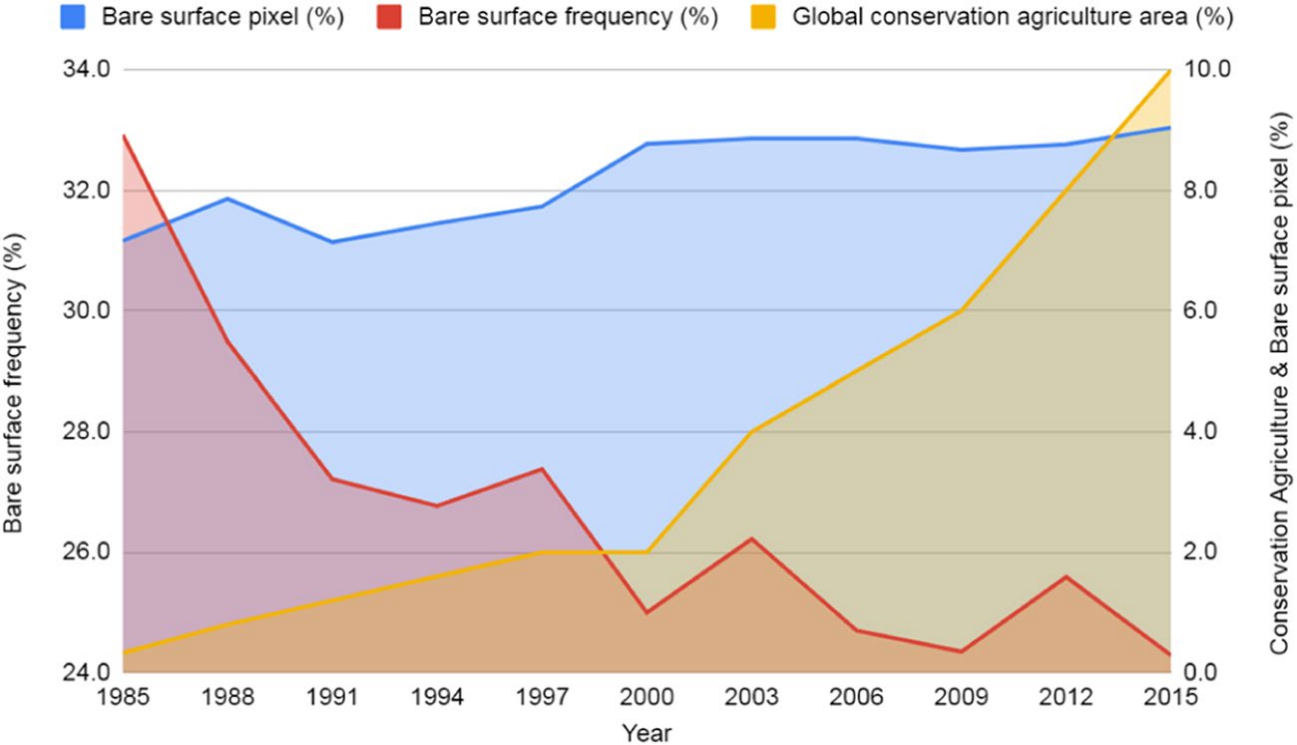
Temporal Earth’s bare surface patterns for each of the three-year periods from 1985 to 2015 in comparison to the worldwide croplands. (Interactive Graph). The global conservation agriculture area (yellow line) was adapted from Kassam *et al*.^48^ and calculated in terms of the percentages of the total area of cropland in the world, i.e., approximately 1.87 billion hectars^50^.

Although soils are being exposed for agricultural purposes (Figs. 2–4)^48,50^, management after deforestation or during crop seasons may have changed due to a decrease in tillage operations. In addition, irrigated agricultural areas have adopted conservation practices. Despite these changes, our results agree with those of Chrysoulakis *et al*.^51^, who stated that albedo changes were consistent with land cover/use changes and were driven by anthropogenic activities. Indeed, North and South America are making important steps in terms of altering soil management practices, with the aim of preserving soil health by increasing the use of no-till systems^52^.

**Figure 5:**
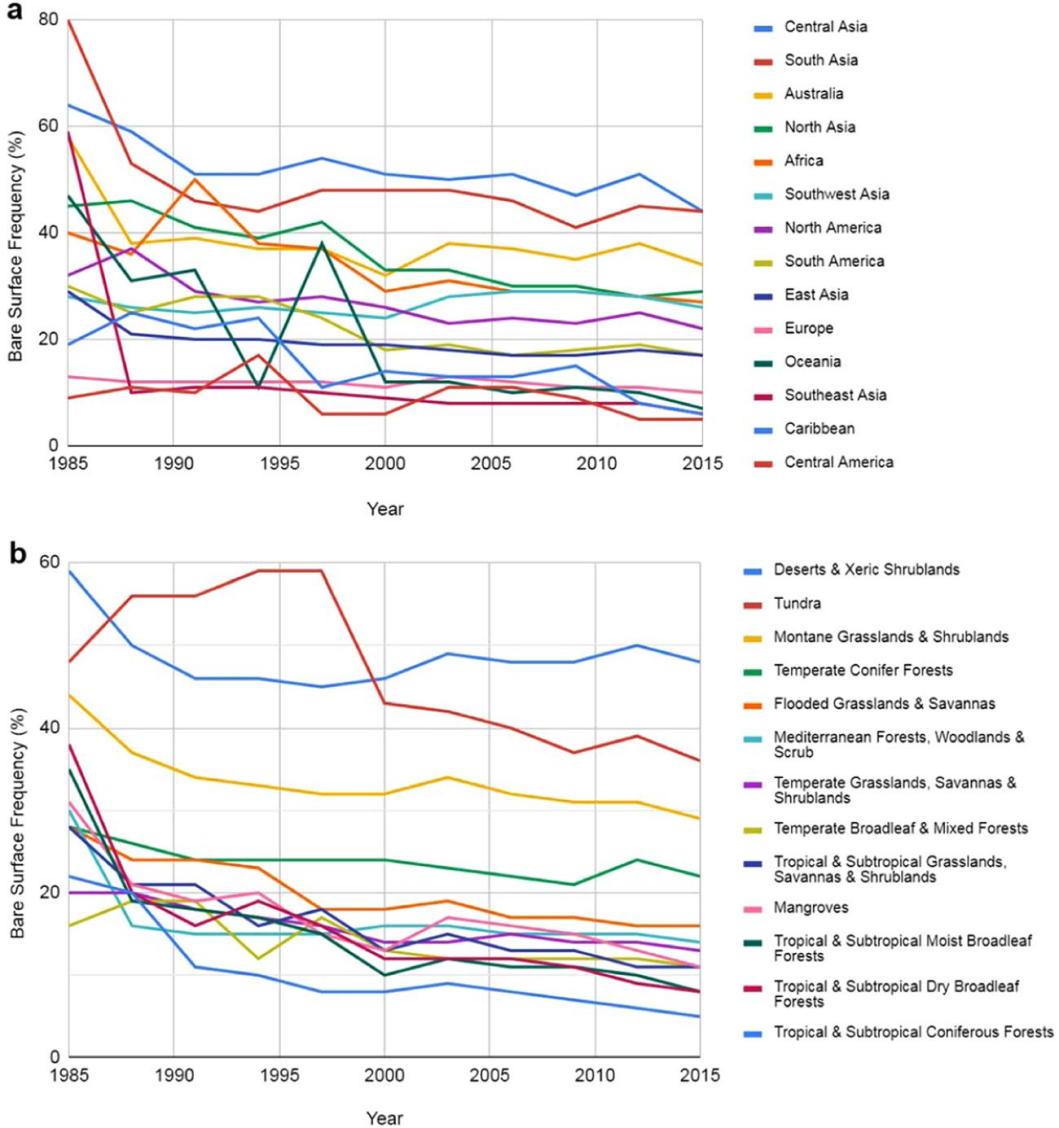
Temporal bare surface frequency from 1985 to 2015. These data present the tendency among bare surface per continent and biomes during the study period: (**a**) continents (Interactive Graph); (**b**) biomes (Interactive Graph).

The situation is also changing in Africa, where the adoption of conservation agriculture (CA) by smallholder farmers in eastern and southern Africa and by medium-scale farmers in northern Africa is increasing^51^. CA in Europe, Russia and Ukraine has been expanding constantly over the past decade, and this change was confirmed by the decrease in the BSF (Fig. 4). These trends are expected to continue^51^. The expansion of the CA system has been especially significant in South America, where Argentina, Brazil, Paraguay and Uruguay are using this system in more than 70% of their total cropland area. South America has 38.7% of the total global area included in CA (63.2% of the cropland in the region), and 35% is found in the United States and Canada (28.1% of the cropland in the region)^51^. Similarly, the BSF presented downward trends for all other regions of the globe (Fig. 5a). The native vegetation showed a stable trend in the BSF over recent years, which agrees with their intrinsic environments (Fig. 5b). Tropical and subtropical areas showed a decrease in the BSF.

Temporal changes in the BSF (Fig. 2) are probably related to patterns previously described in the literature, where satellite data have indicated the greening of Earth^53,54^, with net changes in the leaf area index (LAI) of 5.3 10^6^ km^2^ for the period of 2000–2017. Such an increase is caused by direct factors (e.g., human land-use management) and indirect factors, such as climate change, CO_2_ fertilization effects, nitrogen deposition and recovery from natural disturbances^47^. The main drivers of long-term changes are climate change and CO_2_ fertilization effects^53^. These causes not only affect the LAI but also influence the vegetative development of crops, reducing the exposure frequency of BS in croplands. The short-term changes are most likely related to land management practices. This pattern is clearly observed in China, where ambitious programmes have been implemented to conserve and expand forests, with the goal of mitigating land degradation^55^.

Generally, there is a perception that human activities are exposing soils, leading to degradation^56^. Our results showed that, in general, human activities regarding soil resources are more prone to protect them from degradation. The results are linked with a shift in management, especially no-till practices, with an increase of 1,187% from 1993 to 2013^48^, mainly in Brazil, Argentina, Canada, Australia and China^52^. We also observed that the BST decreased in many countries (Fig. 3), such as in Central Asia, Australia, North America and South America. Approximately 8% of worldwide crops are under no-till conditions^57^, and conservation areas increased 1,607% between 1973 and 1999, reaching 12.5% of the global cropland in 2016^52^. The increase in no-till practices to support the decrease in the BST globally is also indicated by the largest extent of CA adoption, mostly in South and North America, followed by Australia and New Zealand, Asia, Russia and Ukraine, Europe and Africa^52^.

## Limitations

An important step in the methodology is the definition of thresholds for each spectral index (NDVI and NBR2) to discriminate soils from other targets, which usually requires not only expert interpretation of the image but also field observations. Due to the scale of this work, broad thresholds were used to produce the SYSI. Adjustments to classify bare surfaces at the global level might not fully represent the soil spatial variability at regional or local scales. Thus, the coverage and/or frequency of bare surfaces could be increased by adjusting those thresholds according to regional or local spectral patterns. Due to climate variations and moisture conditions, even after a site-specific adjustment of thresholds and temporal averaging, the soil water content might still influence spectral reflectance and soil attribute determination (e.g., clay and SOC contents). If used for spatiotemporal modelling, soil predictions derived from the SYSI reflectance should be carefully evaluated before being applied in decision-making and planning processes.

Another issue is the different frequencies of imagery acquisition and cloud occurrences at the global extent as well as the overlap between scenes that can affect the counting of pixels in the BSF image. Overlapping scenes result in north-south oriented strips in the BSF, which are especially observed in the northern African continent. Such overlapping areas provide little additional information in comparison to fully overlapped images because the images are acquired with minimal time difference and therefore are largely under identical environmental and weather conditions. To tackle these problems, we have generated three different frequency maps regarding the occurrence of 1) total available pixels, 2) cloudless pixels (i.e., flagged as valid for our analysis), and 3) cloudy pixels (Extended Data Fig. 5). Thus, we considered the cloudless density of the globe as the total available pixels for a given location to calculate the relative frequency of bare surfaces instead of their absolute values. The occurrence of cloudy pixels could have hampered the estimates; therefore, the bare surface frequency relied on the number of cloudless pixels for its estimation.

### Concluding remarks

Our processing system was able to retrieve and produce a reasonable estimate of Earth’s surface reflectance. The bare surface showed its potential for monitoring soil resources at the global scale. The SYSI product can assist in the development of public policies and in the assessment of climate alterations, land-use changes and soil management changes. The image presented accurate spectral quality, and the spectral patterns were representative of the abiotic surface materials. The spatial patterns agreed with global datasets, and our results may actually be used to improve them. The global bare surface area increased, while the frequency trend decreased due to the high adoption of agricultural conservation systems. The bare surface reflectance and frequency have a spatiotemporal representation of soil exposure due to natural or human practices, offering interdisciplinary uses such as soil conservation monitoring, soil temperature assessment, precision agriculture, urban expansion, and others. The areas with a low BSF can be associated with lands recently converted to agricultural uses or those with conservation systems in at least the last three decades. Intensive farming during the same time can cause high frequencies of soil exposure. Overall, we observed a negative global tendency of exposed surfaces (−0.16% yr^−1^), corresponding to a reduction in global soil frequency of 4.8% in the last 30 years.

The cloud-based processing environment was efficient by the use of big data, such as the surface reflectance collection. The algorithm took approximately two days to process all the images and produce a single representation of the bare surface for the whole planet. This processing capacity is impressive since the system handles thousands of images simultaneously. If we consider a Landsat image with a native spatial resolution of 30 m, almost one trillion pixels are needed to cover the full extent of Earth. This processing architecture demonstrates the benefits of the availability of open-access satellite images and cloud-based processing systems. This technique enables us to observe the Earth in a timely manner, contributing to soil resource analyses and policy-making. The users can interactively explore the images by accessing the following link: https://geocis.users.earthengine.app/view/bare-surfaces. The interface provides graphical visualization, where charts can be generated by clicking a location over the bare surface areas.

## Methods

### Data acquisition and bare surface retrieval

We used the Google Earth Engine (GEE) cloud platform^58^ to acquire surface reflectance and quality assessment bands of Landsat 4 Thematic Mapper (TM), Landsat 5 TM, Landsat 7 Enhanced Thematic Mapper Plus (ETM+), and Landsat 8 Operational Land Imager (OLI) and to retrieve global bare surfaces from 1985 to 2015. The dataset consisted of higher-level products from the Tier 1 collection, which were processed by the LEDAPS and LaSRC algorithms^59,60^. Once the Landsat bands were positioned in equivalent spectral regions, with slight differences due to their relative spectral responses, we merged the collections by harmonizing the band numbers with a common specific name: blue, green, red, near-infrared (NIR), shortwave-infrared 1 (SWIR_1_), and shortwave-infrared 2 (SWIR_2_).

The Geospatial Soil Sensing System (GEOS3)^23^ algorithm was used to flag bare surfaces (BSs) on images and aggregate the sparse occurrences into a single multispectral representation by the median value. We considered bare surfaces as natural abiotic surfaces (e.g., bare soil, sand and rock outcrops) where natural vegetation was absent or almost absent. In this step, the normalized difference vegetation index (NDVI, Eq. 1), normalized burn ratio (NBR2, Eq. 2) and visible-to-shortwave-infrared tendency index (VNSIR, Eq. 3) were applied in each single image flagging bare surfaces with valid values and other pixel values as not-available (NA) data. Bare surfaces were assigned when the NDVI ranged from −0.25 to 0.25, the NBR2 ranged from −0.30 to 0.10, and the VNSIR was lower than 0.9. Additionally, clouds, shadows and other problematic pixels were removed from the soil classification step using the quality assessment band (pixel_qa) and custom cloud masks of each Landsat sensor.

1 $$NDVI=\frac{(NIR-Red)}{(NIR+Red)}$$

2 $$NB{R}_{2}=\frac{(SWI{R}_{1}-SWI{R}_{2})}{(SWI{R}_{1}+SWI{R}_{2})}$$

3 $$VNSIR=1-[(2\ast Red-Green-Blue)+3(SWI{R}_{2}-NIR)]$$

where Blue, Green, Red, NIR, SWIR1 and SWIR2 are the harmonized spectral bands from Landsat 4 TM, 5 TM, 7 ETM+ and 8 OLI, respectively.

The BS frequency (BSF) was calculated by dividing the number of pixels flagged as bare surface by the number of the same pixels had valid information, i.e., without clouds, shadows or inconsistent values, which were masked using the quality bands. We calculated the BSF for two different time intervals: total (from 1985 to 2015) and every 3 years starting from 1985. The GEOS3 algorithm was implemented in GEE using the JavaScript API. The processing workflow used Landsat products with a native spatial resolution (30 m), and the final global products were resampled and stored at 250 m (Fig. 1).

### Data analysis

#### Quality assessment

We checked the quality of the BS image using the spectral signature and soil line concept^20,23^. The BS spectral signature has a constant ascendant pattern from the blue to SWIR_1_ bands due to the absorption of organic matter and iron oxides in the visible range and a strong reflectance of some minerals (such as quartz) in the near and shortwave-infrared^28,61^. The soil line evaluates the linear relationship (scatterplot) between the red and NIR bands using the slope and offset parameters of the adjusted line^62^. In such cases, the BS has an adjustment near the 1:1 trend line, while vegetation and other land covers have a scattered distribution^18^. For this step, we sampled bare surface pixels inside a 50 km^2^ square using GEE at eight different positions around the world (Fig. 1a–h). Additionally, a median composite (from 2017 to 2019) from the Landsat 8 surface reflectance catalogue was used to compare the bare surface trend with the natural landscape patterns.

#### Statistical correlation between BS image and global maps

Statistical analysis was implemented to quantitatively establish the relationship between the SYSI and the global maps of soil carbon, lithology, and soil classes^63^. For this task, we resampled the global maps and the SYSI to a standard reference grid with a 0.5° pixel resolution. The categorical maps were upscaled using the nearest neighbour method, while maps with continuous information were resampled using the bilinear algorithm. This processing was performed to transform each pixel location (720 × 360 pixels in total) into tabular data for statistical analysis. For the SYSI map, principal component analysis was also performed to combine all the variability of the SYSI bands into single uncorrelated principal components. The first principal component, which explained at least 90% of the variance, was used to determine the strength of association with the global maps.

Two different association methods were implemented considering the continuous and categorical aspects of the maps. The association between the first principal component of the SYSI and the global soil carbon map (both continuous variables) was obtained using Pearson’s correlation analysis. When evaluating the association between the SYSI and the lithological or soil class global maps, one-way analysis of variance (ANOVA) was used. The statistical correlation from ANOVA was calculated using the square root of the ratio between the sum of squares of the model (categorical levels) and the total sum of squares (sum of model with residuals sum of squares)^64^.

#### Temporal analysis

Temporal analysis was performed using the bare surface frequency calculated for each three years from 1985 to 2015. A square grid of 0.1° × 0.1° was created for the global extent (from 180 °W to 180°E and from 70 ° S to 70 ° N), and the three-year frequency of 250 m was reduced by the mean statistics to the extent of 0.1° × 0.1°. For each grid cell, we performed a time series analysis using the Mann-Kendall test (MK)^64,65^. The method defines whether a variable changes consistently over time, revealing an increasing or decreasing trend. The test allows a trend evaluation in normally and non-normally distributed data, which makes it a robust method^66^. The MK test starts by applying an indicator function (*sgn*) on the difference between all possible pairs of measurements (Eq. 4). The value measured at time (*j(x*_*j*_)) is subtracted from the values previously observed (*x*_*i*_), considering that time (*j* > *i*). Then, these differences are used to define Kendall’s statistics (*S*) (Eq. 5).

4 $$sgn(\theta )=\{\begin{array}{cc}+1 & \text{for}\,\theta  < 0\\ 0 & \text{for}\,\theta =0\\ -1 & \text{for}\,\theta  > 0\end{array}\}$$

5 $$S={\sum }_{i=1}^{n-1}{\sum }_{j=i+1}^{n}sgn({x}_{j}-{x}_{i})$$

6 $$V(S)=\frac{1}{18}[n(n-1)(2n+5)]$$

7 $$Z=\left\{\begin{array}{cc}\frac{S-1}{\sqrt{V(S)}} & if\,S > 0\\ 0 & if\,S=0\\ \frac{S+1}{\sqrt{V(S)}} & if\,S < 0\end{array}\right\}$$

where *n* is the length of the dataset. Based on *S*, the variance *V(S)* (Eq. 6) and the normalized test statistics *Z* (Eq. 7) are calculated.

#### Area calculation

The calculation of area was conducted in GEE by reducing the number of pixels inside each country polygon delimited by the large-scale international boundary polygons (simplified) dataset^56^. For this step, a water mask generated by Hansen *et al*.^67^ was used to mask out both oceans and surface water within continents. We counted the total number of pixels (250 m) inside the country boundary as well as the number of bare surface pixels. With the two counts, we calculated the relative area of bare surfaces and the absolute area from the product with the official land area of each country^68^. Additionally, we considered the Moderate Resolution Imaging Spectroradiometer (MODIS) Land Cover Type Early product from 2017 to conduct additional analyses considering different land-use and land cover types, as defined by the Annual International Geosphere-Biosphere Program (IGBP) classification^25^.

## Supplementary information

Supplementary information

## Data Availability

The dataset generated during the current study is available in Google’s Earth Engine App provided in the link: https://geocis.users.earthengine.app/view/bare-surfaces.

## Code availability

The code to generate the final product of the bare surface image is available from the corresponding author upon reasonable request for the following reasons: a) the script/code creates a proxy from which our research group is working on the development of new products that will be published in the near future.
