# Supplementary material for: Bare Earth’s Surface Spectra as a Proxy for Soil Resource Monitoring
Source: Sci Rep. 2020 Mar 10;10:4461. doi: 10.1038/s41598-020-61408-1 (PMC7064585; doi:10.1038/s41598-020-61408-1)
Supplement: Supplementary file 1 — Supplementary information [file 41598_2020_61408_MOESM1_ESM.docx]

**Bare Earth’s Surface Spectra as a Proxy for Soil** **Resource Monitoring**

José A. M. Demattê*^1^, José Lucas Safanelli^1^, Raul Roberto Poppiel^1^, Rodnei Rizzo^1^, Nélida Elizabet Quiñonez Silvero^1^, Wanderson de Sousa Mendes^1^, Benito Roberto Bonfatti^1^, Andre Carnieletto Dotto^1^, Diego Fernando Urbina Salazar^1^, Fellipe Alcântara de Oliveira Mello^1^, Ariane Francine da Silveira Paiva^1^, Arnaldo Barros Souza^1^, Natasha Valadares dos Santos^1^, Cláudia Maria Nascimento^1^, Danilo Cesar de Mello^1^, Henrique Bellinaso^1^, Luiz Gonzaga Neto^1^, Merilyn Taynara Accorsi Amorim^1^, Maria Eduarda Bispo de Resende^1^, Julia da Souza Vieira^1^, Louise Gunter de Queiroz^1^, Bruna Cristina Gallo^1^, Veridiana Maria Sayão^1^, Caroline Jardim Lisboa^1^

^1^ Department of Soil Science, Luiz de Queiroz College of Agriculture, University of São Paulo, Ave Padua Dias 11, Piracicaba, Sao Paulo, Brazil 13418-900

* Corresponding author e-mail: jamdemat@usp.br

**Extended Data**


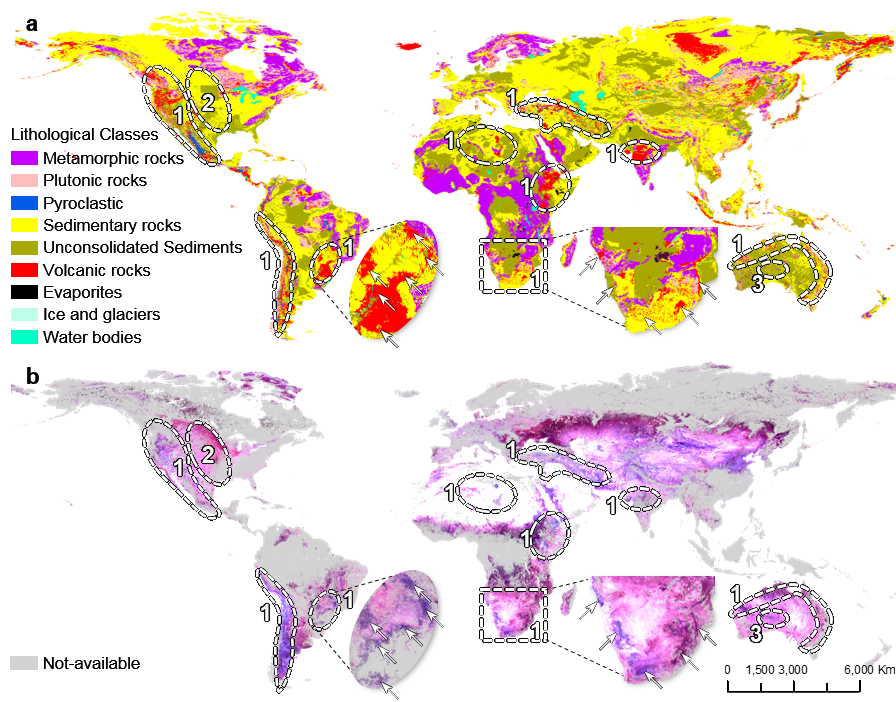


**Extended Data Fig. 1** **Spatial relationship between Earth’s bare surface reflectance and lithology sites.** Each site is described in relation to image spectra and lithology composition (sites 1 to 3). **a)** The global lithologic map^34^; **b)** Earth’s bare surface reflectance in false colour composition (red: 1550-1750 nm, green: 760-900 nm, blue: 630-690 nm). (Landsat 4, 5, 7 and 8 images courtesy of the U.S. Geological Survey).


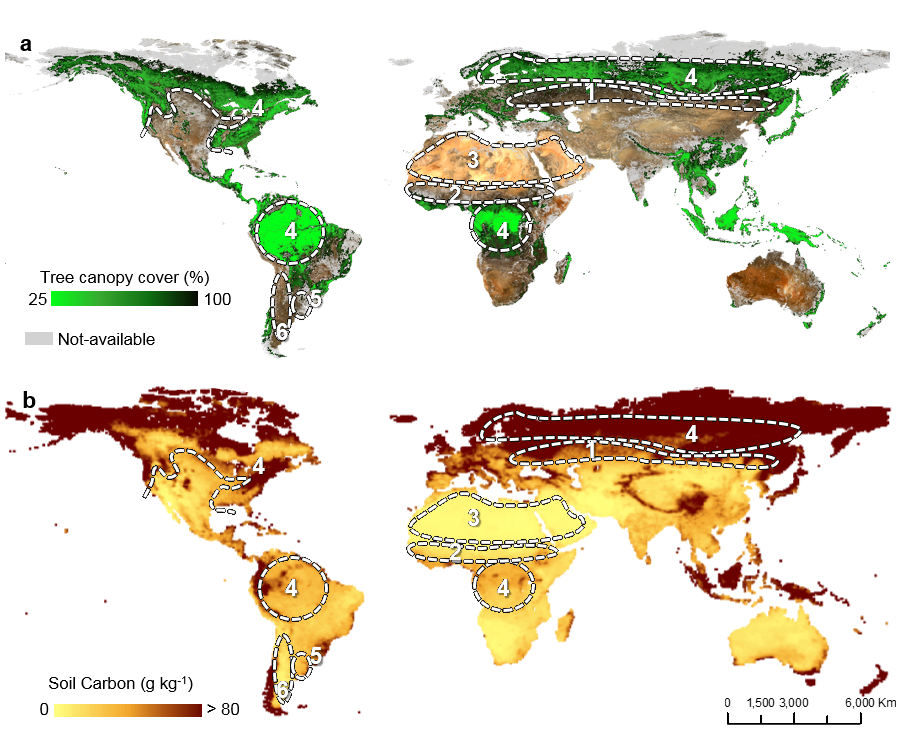


**Extended Data Fig. 2** **Comparison between satellite bare surface images and global carbon maps.** Sites (1 to 4) indicate the relationship between the global soil carbon content and spectral trend of bare surfaces extracted from images: **a)** Global soil organic carbon content (g kg^-1^) from 0-5 cm depth^37^; **b)** Tree canopy cover (> 5 m height) for year 2000^67^ overlapped with bare surfaces in true colour composition (red: 630-690 nm, green: 520-600 nm, blue: 450-520 nm). (Landsat 4, 5, 7 and 8 images courtesy of the U.S. Geological Survey).


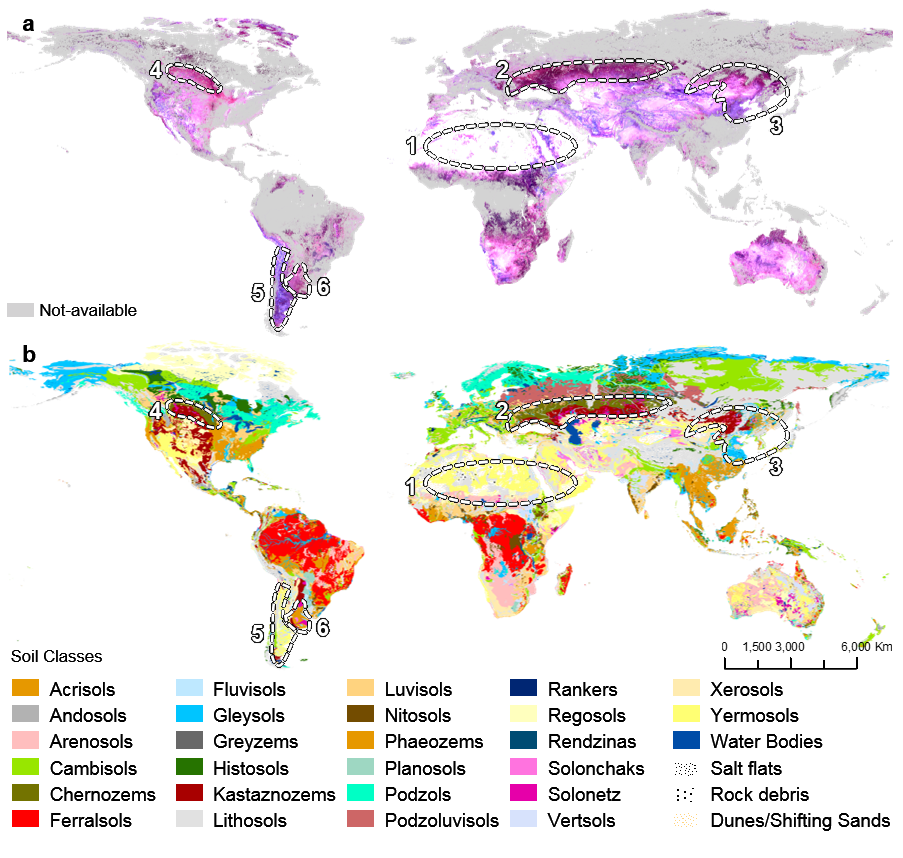


**Extended Data Fig. 3** **Spatial relationship between Earth’s bare surface reflectance and pedological information.** Sites (1 to 3) indicate the image colour and the respective soil classification; **a)** Earth’s bare surface reflectance in false colour composition (red: 1550-1750 nm, green: 760-900 nm, blue: 630-690 nm); **b)** world soil map^63^. (Landsat 4, 5, 7 and 8 images courtesy of the U.S. Geological Survey).


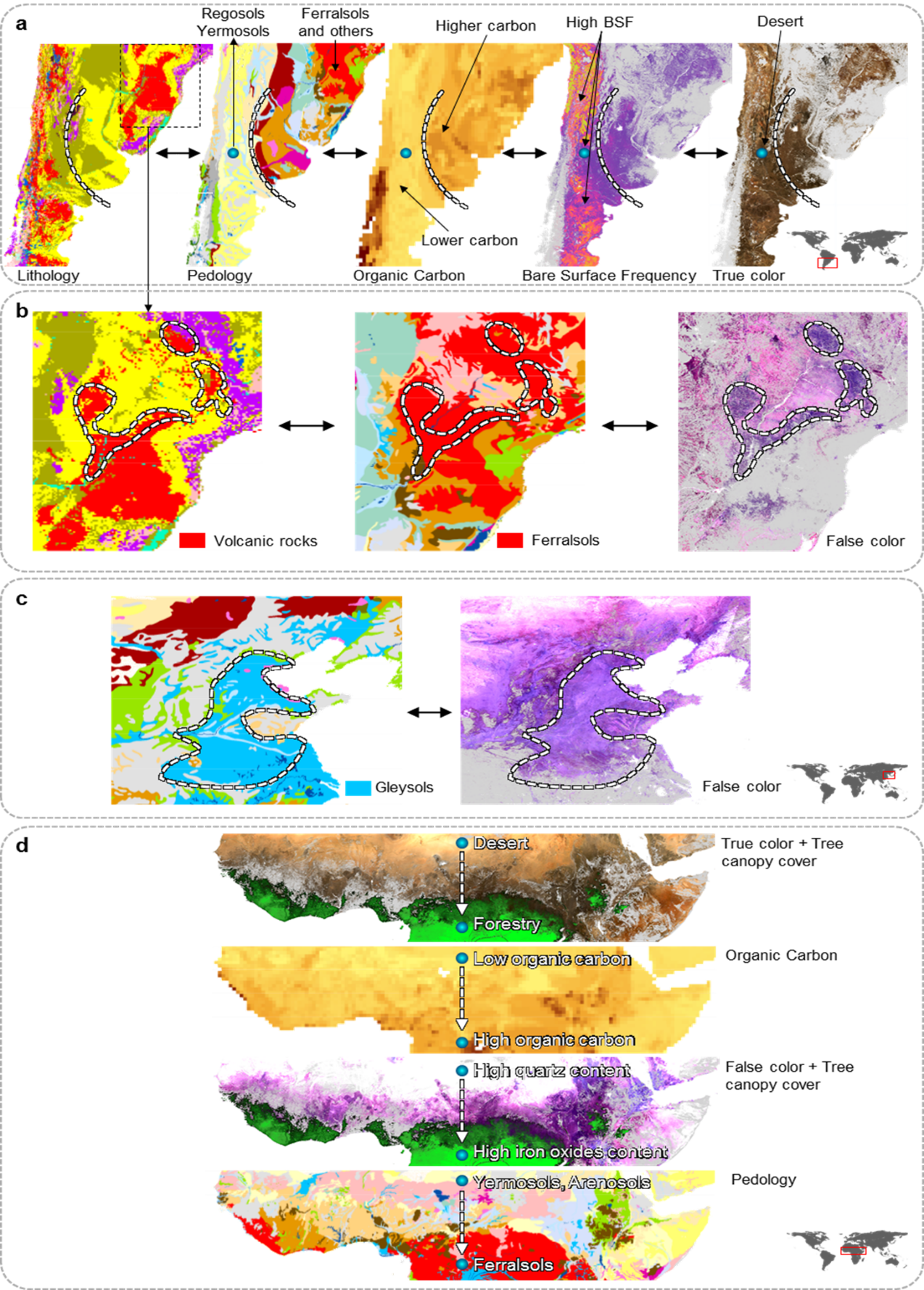


**Extended Data Fig. 4 Linking Earth's bare surface to world maps.** Relationship between lithology^34^, soil classes, organic carbon content^37^, bare surface frequency and bare surface reflectance in true (red: 630-690 nm, green: 520-600 nm, blue: 450-520 nm) and false (red: 1550-1750 nm, green: 760-900 nm, blue: 630-690 nm) colour composition; **a**) South America; **b**) Brazil; **c**) Southern Asia; and **d)** North Africa. (Landsat 4, 5, 7 and 8 images courtesy of the U.S. Geological Survey).


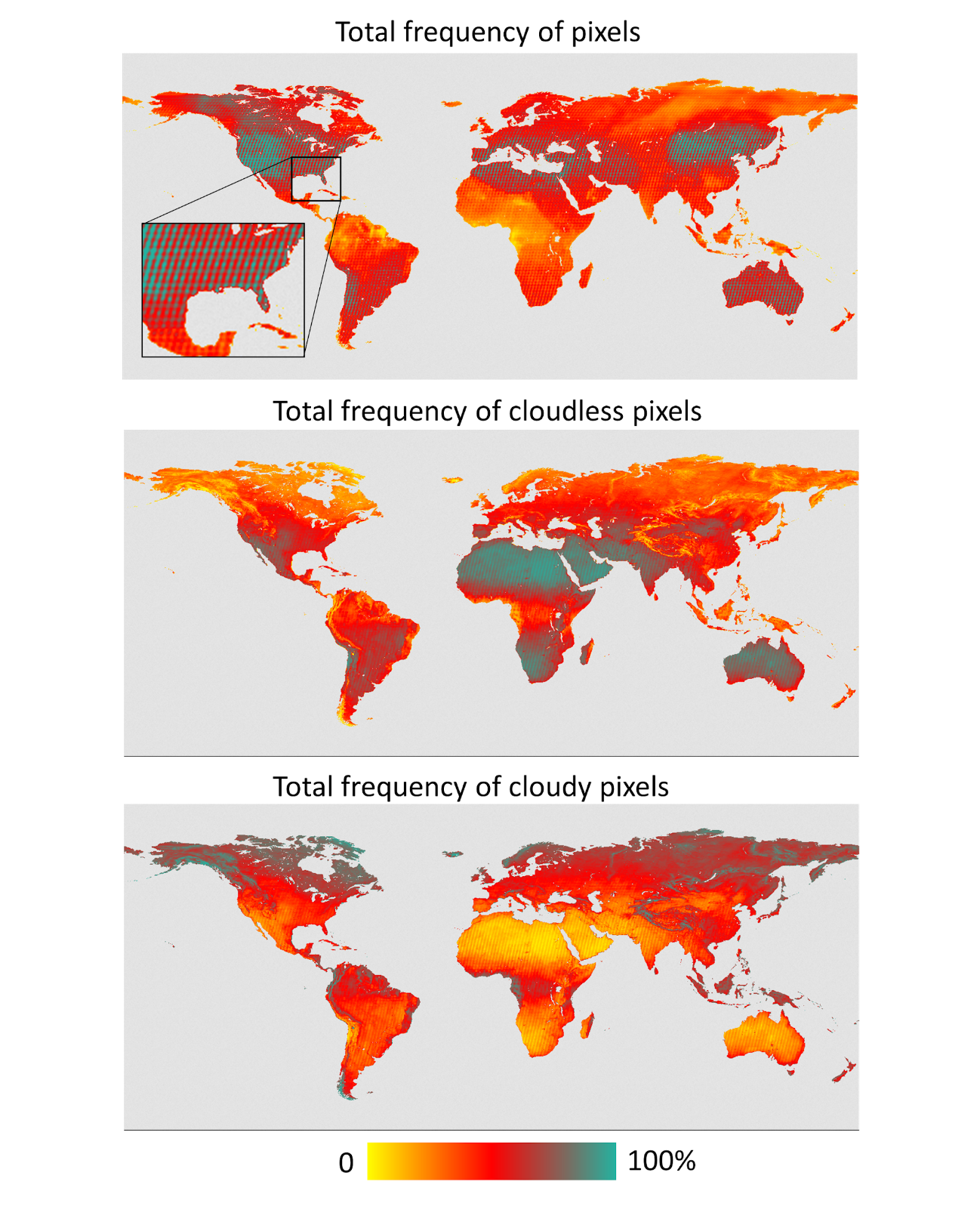
**Extended Data Fig. 5 Cloud frequency maps.** Frequency maps related to how many times the pixel was covered with clouds. (Landsat 4, 5, 7 and 8 images courtesy of the U.S. Geological Survey).
